# 1726. Patterns of Isavuconazole Use for Invasive Fungal Infections

**DOI:** 10.1093/ofid/ofac492.1356

**Published:** 2022-12-15

**Authors:** Vanessa Gow-Lee, Supavit Chesdachai, Jennifer Gile, Nadia Akhiyat, Courtney E Harris, Omar M Abu Saleh

**Affiliations:** Mayo Clinic, Rochester, Minnesota; Mayo Clinic, Rochester, Minnesota; Mayo Clinic, Rochester, Minnesota; Mayo Clinic, Rochester, Minnesota; Brigham & Women's Hospital, Boston, Massachusetts; Division of Public Health, Infectious Diseases, and Occupational Medicine, Department of Medicine, Mayo Clinic, Rochester, Minnesota

## Abstract

**Background:**

Isavuconazole (ISA) is a relatively recent triazole antifungal with several attractive qualities and so increased clinical experience and data on the use of ISA are desirable. However, the data on the pattern of ISA use in invasive fungal infections (IFI) is limited.

**Methods:**

We conducted a retrospective study of patients with probable or proven IFI treated with ISA at Mayo Clinic from 1/1/2015 to 4/1/2020 to examine temporal trends and characteristics of ISA use.

**Results:**

The use of ISA rose over this period, from 4 cases in 2015 to 42 cases in 2019. Of 131 patients identified, ISA was used as the initial antifungal in 15 (11.5%) patients and as the secondary agent in 116 (88.5%) patients. The most cited reasons for choosing ISA as first-line therapy were drug-drug interactions (5, 33%) and baseline QTc prolongation (3, 20%). In cases of ISA used as a secondary agent, the reasons for transitioning to ISA were QTc prolongation (22, 19%), followed by judged failure of the primary therapy (20, 17%), drug-drug interactions (12, 10%), and various toxicities with the first agent. ISA was discontinued early in 81 (62%) patients. Common reasons for early discontinuation were patient death or comfort care (27, 33%), intolerance (18, 22%), failure of therapy (17, 21%), cost or access (6, 7%), drug-drug interaction (3, 4%), issues with absorption or drug delivery (3, 4%), and microbe susceptibility (1, 1%). ISA was judged successful and used as definitive treatment in the remaining 50 (38%) patients. About 30 patients took >1 year of ISA, and no major adverse effects from long-term therapy were noted.

**Conclusion:**

Although ISA is approved for pulmonary aspergillosis and mucormycosis, we found that few patients were treated with ISA as either primary or salvage therapy. In cases where it was used, shortening the QTc and fewer drug-drug interactions were its most attractive qualities. Although it is a newer azole, the morbidity common with other antifungals should prompt more studies on ISA’s efficacy to increase clinicians’ confidence in this option.

**Disclosures:**

**All Authors**: No reported disclosures.

